# The Association Between the Triglyceride–Glucose Index and the Risk of Cardiovascular Disease in Patients with Type 2 Diabetes Mellitus: A Cross-Sectional Study

**DOI:** 10.3390/life15101519

**Published:** 2025-09-26

**Authors:** Munther S. Momani, Dia Sarhan, Zaid Sarhan, Omar R. Aldarras, Raneem Dalaeen, Yazan M. Momani, Khalil S. Mousa, Ahmad Toubasi

**Affiliations:** 1 Department of Internal Medicine, Faculty of Medicine, The University of Jordan, Queen Rania Street, Amman 11942, Jordan; diasarhan2004@gmail.com (D.S.);; 2 Neurology Department, Vanderbilt University Medical Center, Nashville, TN 37235, USA

**Keywords:** triglyceride–glucose index, cardiovascular disease, diabetes mellitus

## Abstract

Objective: This study aimed to evaluate the association between the triglyceride–glucose index (TGI) and the risk of cardiovascular disease in patients with type 2 diabetes mellitus. Methods: This study included 1348 type 2 diabetic patients who attended the endocrinology clinic at Jordan University Hospital between March 2023 and September 2023. Medical records were reviewed to identify patients with documented cardiovascular disease, and the triglyceride–glucose index (TGI) was calculated for each patient. Results: Our results showed that the TGI was higher among patients who developed any major adverse cardiovascular event (MACE), coronary artery disease (CAD), congestive heart failure (CHF), and/or myocardial infarction (MI) compared to those who did not (*p* < 0.001 for all comparisons). Significantly higher TGI values in males were associated with higher odds of a MACE, stroke, and CHF; higher TGI values in females were associated with higher odds of a MACE, CAD, and MI. Higher TGI values in patients younger than 60 were associated with higher odds of a MACE, CAD, CHF, and MI, while in patients older than 60, higher TGI values were associated with higher odds of CAD and MI. The TGI value that was the most predictive of a MACE in our population was >9.36, >9.39 for CAD, and >9.40 and >9.39 for CHF and MI, respectively. Conclusion: The TGI was shown to be associated with a significantly higher risk of a MACE, CAD, CHF, and MI in the whole population, along with stroke in males only. The TGI was more strongly associated with MACEs in patients younger than 60 years compared to older patients. In conclusion, the TGI was associated with cardiovascular outcomes in this diabetic cohort; however, its discriminative ability was modest (AUC 0.55–0.64). This indicates that the TGI alone is insufficient as a predictive tool and should be interpreted alongside established risk factors. Prospective studies are needed to clarify its incremental value in risk prediction.

## 1. Introduction

Cardiovascular diseases (CVDs) are the leading cause of death globally, with an estimated 19.8 million people dying from CVDs in 2022, representing 32% of all global deaths [1]. Furthermore, findings from a comprehensive Global Burden of Disease study covering the years 1990 through 2019 revealed that the burden of CVD, in terms of DALYs (disability-adjusted life years) and deaths, continues to increase globally. The number of prevalent cases of total CVD nearly doubled, from 271 million in 1990 to 523 million in 2019. Additionally, CVD deaths consistently rose, from 12.1 million in 1990 to 18.6 million by 2019. Moreover, global trends for DALYs and years of life lost increased significantly, while years lived with disability doubled [2]. Hypertension, smoking, dyslipidemia, and diabetes are well-established risk factors for developing CVD [3]. Consequently, the early identification of individuals at elevated risk for developing cardiovascular disease is crucial to implement measures to reduce mortality rates and the prevalence of cardiovascular disease.

The triglyceride–glucose index (TGI) is a biomarker that combines fasting triglyceride and fasting glucose levels. Previous research has shown that it serves as an effective alternative measure for detecting insulin resistance (IR) due to its high sensitivity and specificity [4]. The TGI has also been demonstrated to effectively predict adverse clinical outcomes, including coronary artery disease (CAD) [5], heart failure [6], ischemic stroke [7], atherosclerosis [8], non-alcoholic fatty liver disease (NAFLD) [9], and hypertension [10]. While the TGI and its association with major adverse cardiovascular events (MACEs) have been extensively investigated in other regions, there is limited data from the Middle East, making our study the first to address this gap. Importantly, Middle Eastern populations differ from Western and Asian cohorts in the distribution of cardiometabolic comorbidities, genetic susceptibility, and lifestyle-related risk factors. For example, rising obesity prevalence has been well-documented in Middle Eastern and North African (MENA) countries [11], and metabolic syndrome (MetS) has been reported to be highly prevalent among Arabian populations [12]. These differences highlight the importance of investigating the relationship between the TGI and CVD in this unique regional context.

Glucose and lipid metabolism disorders are key drivers in the development of type 2 diabetes mellitus (T2DM) [13]. The TGI has also been shown to demonstrate a strong link to the onset of type 2 diabetes and its complications [4]. CVD accounts for nearly 18.6 million deaths annually. Diabetes is a major contributor to these deaths, underscoring its role in cardiovascular mortality [14]. Approximately 537 million people worldwide between the ages of 20 and 79 were estimated to have diabetes mellitus (DM) in 2021; estimates suggest that number will increase to 643 million by 2030 and 783 million by 2045 [15]. In Jordan, the prevalence of diabetes has shown a significant rising trend, increasing from 14.2% in 1994 to 32.4% by 2017, highlighting the growing burden of the disease in the country [16]. Given this global context and the unique risk factor profile of Middle Eastern populations, our study aims to explore, for the first time, the relationship between the TGI and the risk of cardiovascular disease in patients with diabetes in Jordan.

## 2. Methods

### 2.1. Study Design and Population

A cross-sectional study was conducted at a single tertiary care center, the outpatient endocrinology clinic at Jordan University Hospital (JUH), between March 2023 and September 2023. A total of 1414 T2DM patients, aged between 30 and 84 years, were initially considered for inclusion. Participants were enrolled regardless of the presence or absence of a history of cardiovascular events, including major adverse cardiovascular events (MACEs). All participants provided written informed consent prior to enrollment. Patients were excluded if they had a known advanced malignancy, incomplete clinical data (particularly data required to calculate the triglyceride–glucose index (TGI)), or if they declined consent. Based on these criteria, 66 patients were excluded, resulting in a final sample size of 1348 participants. The study protocol was reviewed and approved by the Institutional Review Board (IRB) of Jordan University Hospital (approval reference number: 10/2025/5593), and all procedures complied with the ethical standards.

### 2.2. Assessment of Triglyceride–Glucose Index

The TGI was computed using the following formula: (Ln [triglycerides (mg/dL) × fasting glucose (mg/dL)/2]) [17]. Both triglyceride and fasting glucose concentrations were determined via an enzymatic assay using an automated biochemistry analyzer (Atellica CI Analyzer, Siemens Healthineers AG, Forchheim, Germany) in our hospital’s central laboratory.

### 2.3. Assessment of Cardiovascular Risk

Cardiovascular risk assessment focused on identifying patients with a documented history of major cardiovascular (CV) comorbidities, including one or more of the following: myocardial infarction (MI), congestive heart failure (CHF), coronary artery disease (CAD), and stroke. For this study, these events were grouped as major adverse cardiovascular events (MACEs).

MI and CAD were defined based on cardiology-confirmed diagnoses, including electrocardiographic changes, elevation of cardiac biomarkers, and/or imaging evidence of coronary artery disease. CHF was defined via echocardiographic findings of reduced ejection fraction or a documented cardiology-confirmed diagnosis of heart failure. Stroke was confirmed via neurology documentation supported by CT or MRI findings.

Only cardiovascular events that occurred within the 10 years preceding enrollment (March 2013–September 2023) were considered.

All classifications relied on the electronic medical record (EMR) system at JUH, including physician notes, laboratory data, and imaging reports. Two trained clinical staff members independently reviewed records to minimize misclassification.

Stroke cases were recorded as a single category without distinction between ischemic and hemorrhagic subtypes, as this level of detail was inconsistently available in the EMRs.

Patients with a confirmed diagnosis of any of these conditions were classified as having a MACE and were included in the relevant subgroup analysis.

### 2.4. Assessment of Diabetes

DM was defined as either being on pharmacological treatment for diabetes mellitus or having a diagnosis with hemoglobin A1c ≥ 6.5%, fasting plasma glucose (FPG) ≥ 126 mg/dL, or a 2 h blood glucose ≥ 200 mg/dL, consistent with the American Diabetes Association guidelines [18].

### 2.5. Section of Covariates

Data on various demographic and health-related factors were collected through the medical record system at Jordan University Hospital by trained clinical staff. The collected covariates included age, gender, duration of diabetes, presence of diabetic complications (such as neuropathy, nephropathy, and retinopathy), smoking status, body weight, body mass index (BMI), and systolic and diastolic blood pressure. Laboratory measurements included hemoglobin A1c, serum creatinine, low-density lipoprotein cholesterol (LDL-C), high-density lipoprotein cholesterol (HDL-C), triglycerides, vitamin D, parathyroid hormone, serum calcium, serum phosphorus, albumin, alkaline phosphatase, and magnesium. Renal function was assessed using the estimated glomerular filtration rate (eGFR), calculated through the Chronic Kidney Disease Epidemiology Collaboration (CKD-EPI) and Modification of Diet in Renal Disease (MDRD) equations. Additionally, medication history was documented, including the use of anti-diabetic agents (metformin, insulin, and other oral hypoglycemics), aspirin, angiotensin receptor blockers (ARBs), angiotensin-converting enzyme inhibitors (ACEIs), statins, beta blockers, diuretics, calcium channel blockers (CCBs), and proton pump inhibitors (PPIs).

### 2.6. Statistical Analysis

Categorical variables were presented using counts and percentages, while continuous variables were presented using means and standard deviations. The Chi-square test and *t*-test were used to determine the association between categorical and continuous variables, respectively, and cardiovascular events.

Regression analysis was used to evaluate the association between the TGI and CV events while adjusting for confounding variables. The partially adjusted model included sex, age, body mass index, and smoking status. The fully adjusted model included the partially adjusted variables along with hypertension, creatinine, systolic and diastolic blood pressure, parathyroid hormone, calcium, albumin, HbA1c, low-density lipoprotein, high-density lipoprotein, insulin, aspirin, angiotensin receptor blockers, angiotensin-converting enzyme inhibitors, statins, beta blockers, diuretics, metformin, calcium channel blockers, proton pump inhibitors, and oral anti-diabetic medication. Analysis for Variance Inflation Factor (VIF) was performed to ensure the stability of the interpretation and associations derived from the fully adjusted model. VIF values were calculated and were all below 5, which was not concerning. Subsequent subgroup analysis was conducted according to sex (males vs. females) and age (<60 years vs. ≥60 years). False discovery rate (FDR) *p*-values were reported for subgroup analyses to correct for multiple comparisons. The odds ratio (OR) and its associated 95% confidence interval (95%-CI) were reported.

Receiver operating characteristics (ROC) analysis was used to determine the optimal cut-off points for the TGI. Sensitivity, specificity, and area under the curve (AUC) values were reported. IBM SPSS v.25 was used to conduct the analysis. A *p*-value < 0.050 was considered statistically significant for all analyses.

## 3. Results

### 3.1. Characteristics of Included Patients

A total of 1348 participants were included in the final analysis, of whom 22.2% had experienced a MACE (299/1348). A detailed look at those who had any MACE outcome reveals that 6.8% developed stroke, 10.8% developed CAD, 9.6% developed CHF, and 11.2% developed MI, as some individuals had more than one complication. Supplementary Table S1 demonstrates the characteristics of the included cohort. Table 1 demonstrates the characteristics of participants according to their MACE status. Males (31.02%) were more likely to develop a MACE compared to females (15.88%) (*p* < 0.001). Diabetes duration was higher among patients with a MACE compared to those who did not (*p* < 0.001). Smoking and hypertension were both associated with MACEs (*p* < 0.001). Higher average creatine (*p* = 0.012) and diastolic blood pressure (*p* < 0.001) were also significantly associated with MACEs. The average glomerular filtration rate was estimated using the MDRD and CKD-EPI equations, and scores were lower among patients with a MACE (*p* < 0.001). HbA1c (*p* = 0.001) and PTH (*p* = 0.008) were higher, while HDL (*p* < 0.001) and calcium (*p* = 0.001) were lower among patients with a MACE. Albumin (*p* < 0.001) was lower while alkaline phosphatase (*p* = 0.031) was higher among patients with a MACE. The use of insulin (*p* = 0.010), aspirin (*p* < 0.001), angiotensin-converting enzyme inhibitors (*p* = 0.002), statins (*p* = 0.002), beta blockers (*p* < 0.001), diuretics (*p* < 0.001), metformin (*p* < 0.001), calcium channel blockers (*p* = 0.001), and proton pump inhibitors (*p* < 0.001) was higher among patients with a MACE.

Supplementary Tables S1–S4 demonstrate the distribution of the patients’ characteristics according to MACE components: stroke, CAD, CHF, and MI, respectively.

### 3.2. Triglyceride–Glucose Index and Cardiovascular Events

*T*-test analysis demonstrated that the TGI was higher among patients who developed a MACE, CAD, CHF, or MI compared to those who did not (*p* < 0.001 for all comparisons). There was no difference in the TGI between patients with and without stroke (*p* = 0.106) (Table 2).

The unadjusted model (Table 3) demonstrated that a higher TGI was associated with higher odds of a MACE (OR = 1.80; 95%CI: 1.48–2.19), CAD (OR = 2.01; 95%CI: 1.56–2.60), CHF (OR = 1.66; 95%CI: 1.27–2.17), and MI (OR = 2.09; 95%CI: 1.63–2.69). Partially and fully adjusted models also showed significant associations between a higher TGI and a MACE, CAD, CHF, or MI.

Analysis per quartiles demonstrated that the third and fourth quartiles were associated with higher odds of a MACE compared to the first quartile in the unadjusted model. Similar results were obtained among the components of MACEs, including CAD, CHF, and MI. Table 4 demonstrates the detailed results of this analysis.

### 3.3. Triglyceride–Glucose Index Performance

**Figure 1:**
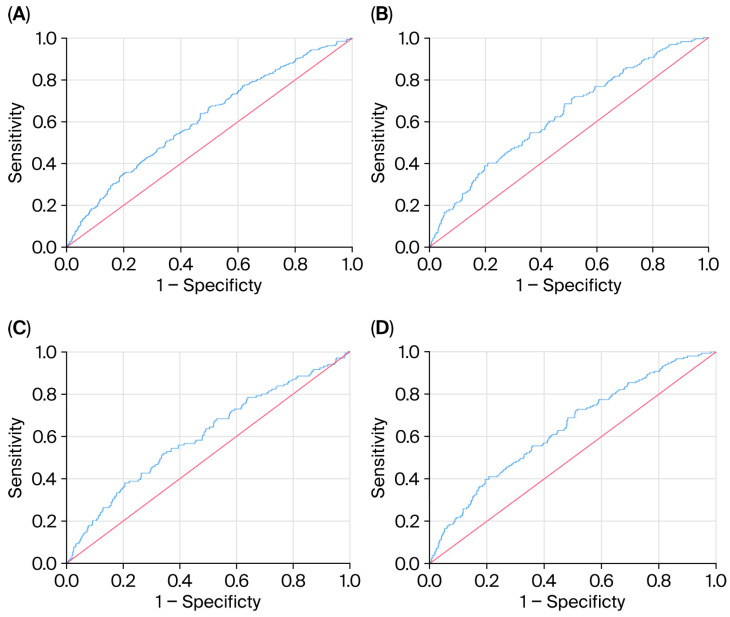
(**A**–**D**) ROC analysis for highest TGI AUC for MACE.

ROC analysis demonstrated that TGI AUC for MACE was the highest using a cut-off > 9.36 (Figure 1A: AUC = 0.61, *p* < 0.001). the TGI AUC for CAD was the highest using a cut-off > 9.39 (Figure 1B: AUC = 0.63, *p* < 0.001). The cut-off points that achieved the highest performance for CHF (Figure 1C: AUC = 0.60, *p* < 0.001) and MI (Figure 1D: AUC = 0.64, *p* < 0.001) were >9.40 and >9.39, respectively. The sensitivity, specificity, and AUC values are described in detail in Table 5.

### 3.4. Subgroup Analysis

Subgroup analysis among males demonstrated that a higher TG-Glu index was associated with higher odds of a MACE (OR = 2.57; 95%CI: 1.29–5.12), stroke (OR = 2.47; 95%CI: 1.08–5.65), and CHF (OR = 3.06; 95%CI: 1.30–3.86). After correction for multiple comparisons, the TG-Glu index remained associated with MACEs (FDR-*p* = 0.031) and CHF (FDR-*p* = 0.046). In contrast, subgroup analysis among females demonstrated that a higher TG-Glu index was associated with higher odds of a MACE (OR = 1.98; 95%CI: 1.43–2.53), CAD (OR = 3.44; 95%CI: 2.62–4.26), and MI (OR = 4.21; 95%CI: 3.39–5.03). After correction for multiple comparisons, the TG-Glu index remained associated with the same outcomes.

Subgroup analysis among patients younger than 60 demonstrated that a higher TG-Glu index was associated with higher odds of a MACE (OR = 3.52; 95%CI: 2.58–4.46), CAD (OR = 5.28; 95%CI: 3.89–6.67), CHF (OR = 4.27; 95%CI: 2.95–5.65), and MI (OR = 7.26; 95%CI: 5.83–8.69). After correction for multiple comparisons, the TG-Glu index remained associated with CAD (FDR-*p* = 0.042) and MI (FDR-*p* = 0.033).

Among patients older than 60, a higher TG-Glu index was associated with higher odds of CAD (OR = 1.91; 95%CI: 1.30–2.52) and MI (OR = 1.82; 95%CI: 1.23–2.41). After correction for multiple comparisons, the TG-Glu index was not associated with any of the aforementioned outcomes. These subgroup findings should be considered exploratory and interpreted with caution. they serve as hypothesis-generating results that require validation in future prospective cohorts.

Table 6 demonstrates the detailed results of subgroup analysis according to age and sex.

## 4. Discussion

The prevalence of metabolic syndrome in Jordan remains alarmingly high, as previously documented by Obeidat et al. [19]. Furthermore, data from the PACT-MEA study reported a substantial burden of atherosclerotic cardiovascular disease (ASCVD) among patients with type 2 diabetes in Jordan [20]. Given the well-established relationship between diabetes and cardiovascular disease (CVD), there is a growing need for simple and effective biomarkers to better stratify cardiovascular risk in diabetic populations.

Our data showed that the TGI was significantly higher in patients who developed major adverse cardiovascular events (MACEs) compared to those who did not, which is consistent with previous work [4,5,6,7,8]. We further analyzed the association of the index and its utility in different age, gender groups, and in individual MACE variables.

### 4.1. TG Index and Age

In our cohort, higher triglyceride–glucose index (TGI) values were observed among both younger (<60 years) and older (≥60 years) participants with major adverse cardiovascular events (MACEs), with stronger associations noted in the younger group (OR = 3.52 vs. 1.53). Baseline characteristics may partly explain this pattern: younger adults had higher HbA1c (7.91 ± 1.72 vs. 7.54 ± 1.46%), a greater prevalence of current smoking (22.6% vs. 12.5%, *p* < 0.001), and slightly lower HDL (44.1 ± 21.2 vs. 45.7 ± 19.9 mg/dL). ROC analysis also suggested that thresholds above 9.36 were linked with a MACE, although discriminative performance was modest (AUC 0.60–0.64).

Comparable trends have been described in other populations. An Iranian study reported stronger associations between the TGI and CVD incidence in younger adults [21]. U.S. data have similarly indicated that the TGI is linearly related to mortality risk in younger cohorts, whereas in older individuals, the association appeared weaker or U-shaped [22,23,24].

Overall, these findings suggest that age may influence how the TGI relates to cardiovascular outcomes, with younger patients showing more consistent patterns of association. However, the observed links remain modest, and in older adults, the relationship is less clear. Given the cross-sectional nature of our study, these results should be interpreted cautiously and validated in prospective settings before being considered for clinical application.

### 4.2. TG Index and Gender

In our fully adjusted sex-stratified models, a higher TGI remained associated with major adverse cardiovascular events (MACEs) and congestive heart failure (CHF) among men (false discovery rate *p* = 0.031 and 0.046, respectively), and with MACEs, coronary artery disease (CAD), and myocardial infarction (MI) among women (FDR *p* = 0.044, 0.015, and 0.006, respectively).

To contextualize these findings, our cohort data revealed that women had a substantially higher BMI compared to men (34.3 vs. 30.8 kg/m^2^), whereas age and diabetes duration were nearly identical between the two groups. In a large Thai cohort of type 2 diabetes patients, Lertsakulbunlue et al. [25] found that the TGI was significantly associated with predicted 10-year cardiovascular risk among both males and females, with very similar effect sizes and modest discriminative ability (AUC ≈ 0.57) between sexes. This aligns with our observation that the TGI is associated with CVD outcomes in both sexes. However, our results should be interpreted with caution as they are exploratory in nature and require validation in future prospective studies.

### 4.3. TG Index and Heart Failure

In our diabetic cohort, patients who experienced congestive heart failure (CHF) had significantly higher TGI values compared to those without CHF (mean: 9.52 vs. 9.29; *p* < 0.001). In unadjusted and partially adjusted models, this association persisted; however, after full adjustment for age, sex, BMI, hypertension, HbA1c, creatinine, and lipid parameters, the relationship attenuated and was no longer statistically significant (*p* = 0.076). This suggests that while the TGI may be related to CHF risk, the association in our cohort is less stable and may have been influenced by confounding and limited statistical power.

Our findings are directionally consistent with a recent analysis of U.S. National Health and Nutrition Examination Survey (NHANES) data (2007–2018), which reported a significant independent association between a higher TGI and incident heart failure in the general population [26]. Mechanistically, insulin resistance has been implicated in pathways contributing to heart failure, including chronic hyperinsulinemia, impaired glucose utilization, dyslipidemia, and myocardial fibrosis, all of which may predispose individuals to CHF in high-risk groups [27]. Validation in larger diabetic cohorts is needed to clarify whether the TGI independently predicts CHF risk after full covariate adjustment.

### 4.4. TG Index and Stroke Risk: Focus on Ischemic Stroke

Our data did not demonstrate a statistically significant association between a higher TGI and stroke incidence. Although patients with stroke exhibited slightly elevated TGI values (mean: 9.42 ± 0.65) compared to those without stroke (mean: 9.30 ± 0.67), this difference did not reach significance (*p* = 0.106). Similar results were observed across our partially and fully adjusted models. It is important to note that our analysis grouped both ischemic and hemorrhagic stroke subtypes together, which may have attenuated any potential associations.

Several studies with large cohorts, such as the Kailuan Study, a study on rural Chinese populations, the ARIC Study, and meta-analyses [28,29,30,31,32] have reported a positive association between the TGI and ischemic stroke, but not hemorrhagic stroke. Our lack of significant findings could be due to the absence of subgroup analyses by stroke subtype and the relatively low number of stroke events within our cohort.

### 4.5. Optimal Cut-Off Value of TG Index for Predicting CV Events and Global Perspective

Our data showed that the TGI threshold most strongly associated with the risk of developing a MACE in patients with type 2 diabetes was >9.36, based on ROC analysis (AUC = 0.61). Similarly, the optimal cut-off values were >9.39 for both CAD and MI, and >9.40 for CHF. These relatively high thresholds may reflect the greater metabolic burden among diabetic patients. Despite Jordan’s high burden of CVD and diabetes, no published studies to date have examined TGI performance in Jordanian or Middle Eastern cohorts, highlighting a key research gap that our study begins to address.

Comparable thresholds have been reported in studies on similar populations. For example, Wang et al. identified a cut-off of 9.323 for predicting 3-year MACEs in diabetic patients with acute coronary syndrome [33]. In contrast, Zhu et al. reported a lower threshold of 8.83 in diabetic patients’ post-percutaneous coronary intervention (PCI), yielding higher sensitivity but lower specificity [34]. On the other hand, the Brazilian PROCARDIO-UFV Study identified a lower cut-off of 9.04 for predicting intermediate/high cardiovascular risk in adults with cardiometabolic features [35]. Similarly, in the Spanish VMCUN cohort, increased cardiovascular risk was observed at TGI levels corresponding to the fourth and fifth quintiles, which translated to a lower average threshold compared to ours [36].

Meanwhile, in Italy, elevated TGI values were linked to increased carotid intima-media thickness in primary prevention populations, although associations with plaque formation varied [37].

Data from Sweden suggest that higher TGI values are associated with increased arterial stiffness—measured by carotid–femoral pulse wave velocity—and are predictive of elevated cardiovascular risk, including cardiovascular mortality. In two large prospective cohorts (Malmö Diet and Cancer Study–Cardiovascular Cohort and Malmö Preventive Project), participants in the highest TGI quartile faced a 37% greater risk of cardiovascular death compared to the lowest quartile (HR 1.37, 95%CI: 1.26–1.49) [38].

Limited data is available from the Middle East. The Tehran Lipid and Glucose Study in Iran concluded that a higher TGI is significantly associated with increased risk of CVD/CHD incidence; this association was more prominent among the younger population [18].

In Asia, an Eastern Chinese cohort showed that the TGI significantly improved long-term CVD risk prediction compared to traditional models, particularly among patients in the highest quartile [28]. Though not directly comparable, Korean pediatric studies linked the TGI to early metabolic abnormalities; however, adult studies in Korea align more closely with diabetes-driven cardiovascular outcomes [39,40].

These differences may reflect population-specific factors, including the prevalence of type 2 diabetes, background cardiovascular risk, and metabolic profile. Moreover, the relatively higher thresholds we observed compared to Asian and Latin American cohorts [33,34,35] may be attributable to earlier diabetes onset, higher obesity rates, and adverse lipid profiles characteristic of Jordanian populations [17,18]. Our findings reinforce the notion that cut-off values derived from non-Middle Eastern or non-diabetic cohorts may underestimate cardiovascular risk in diabetic populations from regions with a high metabolic burden.

Collectively, these results underscore the importance of generating locally derived data and establishing regionally calibrated thresholds prior to clinical adoption, particularly in underrepresented regions like the Middle East and North Africa. It should be mentioned, however, that the cut-off values reported in our study are still exploratory and not validated yet for clinical application at this time, as more local and regional data are needed.

### 4.6. Addressing the Middle Eastern Evidence Gap

Our study provides one of the first large-scale assessments of the TGI in a Middle Eastern diabetic population, helping to address a critical regional research gap. Moreover, this study is novel as it provides locally derived TGI cut-off values and evaluates age- and sex-specific associations in a Middle Eastern cohort, offering insights not previously reported in other populations.

Furthermore, our suggested optimal TGI cut-off for MACE association likely reflects the elevated cardiometabolic burden in Jordanian populations. The interaction between the TGI and locally prevalent risk factors, such as high smoking rates and low HDL-C levels, may amplify the index’s association value beyond insulin resistance alone.

From a health systems perspective, the TGI offers a low-cost, easily calculated screening tool (AUC = 0.61–0.64 for CVD outcomes in our study) that can be easily implemented in resource-limited Jordanian clinics. Its integration into national guidelines could improve early cardiovascular risk detection among high-risk diabetic patients, aligning with the World Health Organization (WHO) recommendations for low- and middle-income countries [41].

Our study also lays the groundwork for broader application in the MENA region. Given the similarity in metabolic risk profiles across countries like Saudi Arabia and Egypt, these findings may inform regional screening strategies and stimulate further validation studies in Arab populations. While these results contribute valuable regional evidence, the cross-sectional design limits causal inference due to a lack of temporality and potential reverse causation, highlighting the need for regional prospective validation studies.

## 5. Conclusions

Cardiovascular disease remains a pressing global health challenge, with diabetes and prediabetes markedly increasing its risks. The triglyceride–glucose index (TGI) is an accessible surrogate for insulin resistance that has shown associations with cardiovascular outcomes in our Jordanian cohort of adults with T2DM. Importantly, while the TGI was significantly associated with MACEs, CAD, MI, and CHF in unadjusted and partially adjusted models, its discriminative performance was modest (AUC 0.55–0.64). This likely reflects the multifactorial nature of cardiovascular disease and the fact that TGI captures only one aspect of metabolic risk. Accordingly, the TGI should be considered a supportive rather than a standalone tool, with potential value when combined with traditional risk factors in multivariable risk models.

The concerning rise in diabetes and CVD prevalence in Jordan and the broader region underscores the urgent need for locally driven research. By providing cohort-specific evidence on the relationship between the TGI and CVD in Jordanian adults with T2DM, this study contributes to clarifying an important knowledge gap and may help guide targeted strategies to reduce cardiovascular complications in high-risk populations. Prospective studies are needed to validate these findings and assess the incremental predictive and clinical utility, and the optimal cut-off values of the TGI in regional and global contexts.

## Figures and Tables

**Table 1:** Characteristics of the included patients according to their MACE status.

	[ALL]	No MACE	MACE	*p* Overall
	*N* = 1412	*N* = 1098	*N* = 314	
Age	60.0 [54.0; 68.0]	59.0 [52.0; 66.0]	64.0 [57.0; 72.0]	<0.001
Gender:				<0.001
0	832 (58.9%)	699 (63.7%)	133 (42.4%)	
1	580 (41.1%)	399 (36.3%)	181 (57.6%)	
DM: Y	1412 (100%)	1098 (100%)	314 (100%)	
HTN:				<0.001
N	432 (30.6%)	387 (35.2%)	45 (14.3%)	
Y	980 (69.4%)	711 (64.8%)	269 (85.7%)	
Weight	84.0 [74.0; 96.8]	83.0 [74.0; 96.0]	88.0 [77.6; 98.0]	0.002
Height	1.61 [1.55; 1.70]	1.60 [1.54; 1.68]	1.65 [1.57; 1.71]	<0.001
BMI	32.0 [28.2; 36.4]	32.0 [28.2; 36.6]	32.0 [28.3; 36.1]	0.875
serum Cr. 1	0.76 [0.60; 1.00]	0.72 [0.58; 0.92]	0.95 [0.71; 1.29]	<0.001
serum Cr2	0.77 [0.60; 1.00]	0.73 [0.58; 0.93]	0.94 [0.73; 1.31]	<0.001
Mean GFR(MDRD)	96.9 [68.1; 123]	102 [74.8; 127]	75.9 [54.0; 103]	<0.001
CKD-EPI 1	91.4 [66.9; 103]	93.8 [74.5; 104]	72.6 [52.0; 95.3]	<0.001
HBAic < 7, 7–8, 8–9, >9%	7.40 [6.50; 8.60]	7.30 [6.50; 8.50]	7.90 [6.70; 9.00]	0.001
LDL > 100	92.0 [72.6; 114]	92.0 [73.0; 114]	88.0 [71.0; 114]	0.248
HDL < 38 men, <50 women	43.0 [36.0; 51.0]	44.0 [36.0; 52.0]	39.0 [33.0; 47.0]	<0.001
Trig > 160	147 [108; 208]	144 [107; 203]	154 [120; 232]	0.003
Vit d 1	19.2 [12.3; 29.2]	19.3 [12.3; 29.4]	18.9 [12.3; 29.0]	0.873
vit d 2	30.3 [18.9; 45.2]	30.8 [19.1; 45.4]	27.6 [17.2; 42.9]	0.307
Vit_d_mean	24.3 [16.1; 35.9]	24.7 [16.6; 35.9]	23.5 [15.7; 35.4]	0.277
serum_Cr_mean	0.76 [0.60; 1.00]	0.72 [0.58; 0.92]	0.94 [0.74; 1.27]	<0.001
Mean_GFR_MDRD_mean	97.1 [69.4; 121]	102 [77.2; 125]	77.5 [56.7; 105]	<0.001
CKD_EPI_mean	92.1 [68.8; 103]	94.8 [76.1; 106]	76.4 [54.0; 95.2]	<0.001
Smoking (never/current smoker/ex-smoker):				<0.001
N/N/N	1 (0.07%)	1 (0.09%)	0 (0.00%)	
N/N/Y	177 (12.5%)	106 (9.65%)	71 (22.6%)	
N/Y/N	27 (1.91%)	19 (1.73%)	8 (2.55%)	
N/Y/Y	213 (15.1%)	155 (14.1%)	58 (18.5%)	
N/YY	2 (0.14%)	2 (0.18%)	0 (0.00%)	
Y/N/N	991 (70.2%)	815 (74.2%)	176 (56.1%)	
Y/NN	1 (0.07%)	0 (0.00%)	1 (0.32%)	
PTH	69.9 [48.4; 109]	65.9 [47.0; 99.6]	88.9 [53.0; 137]	<0.001
Ca	9.50 [9.20; 9.80]	9.52 [9.24; 9.80]	9.40 [9.10; 9.70]	<0.001
Phosphorous	3.50 [3.10; 3.90]	3.50 [3.10; 3.90]	3.40 [3.00; 3.86]	0.080
Albumin	4.32 [4.10; 4.53]	4.34 [4.14; 4.55]	4.26 [3.99; 4.48]	<0.001
Alkaline phosph.	79.0 [64.0; 97.0]	79.0 [64.0; 94.0]	83.0 [64.0; 104]	0.038
Mg	1.80 [1.60; 1.92]	1.80 [1.60; 1.91]	1.80 [1.60; 1.92]	0.612
Insulin:				0.010
N	712 (50.5%)	575 (52.5%)	137 (43.6%)	
Y	698 (49.5%)	521 (47.5%)	177 (56.4%)	
Aspirin:				<0.001
N	415 (29.4%)	368 (33.6%)	47 (15.0%)	
Y	995 (70.6%)	728 (66.4%)	267 (85.0%)	
ARBs:				0.039
N	923 (65.5%)	734 (67.0%)	189 (60.2%)	
Y	487 (34.5%)	362 (33.0%)	125 (39.8%)	
ACEIs:				0.005
N	1147 (81.3%)	910 (83.0%)	237 (75.5%)	
Y	263 (18.7%)	186 (17.0%)	77 (24.5%)	
Statins:				0.002
N	247 (17.5%)	212 (19.3%)	35 (11.1%)	
Y	1163 (82.5%)	884 (80.7%)	279 (88.9%)	
Beta Blockers:				<0.001
N	791 (56.1%)	703 (64.1%)	88 (28.0%)	
Y	619 (43.9%)	393 (35.9%)	226 (72.0%)	
Diuretics:				<0.001
N	898 (63.7%)	759 (69.3%)	139 (44.3%)	
Y	512 (36.3%)	337 (30.7%)	175 (55.7%)	
Metformin:				<0.001
N	328 (23.3%)	228 (20.8%)	100 (31.8%)	
Y	1082 (76.7%)	868 (79.2%)	214 (68.2%)	
CCBs:				0.002
N	1009 (71.6%)	808 (73.7%)	201 (64.0%)	
Y	401 (28.4%)	288 (26.3%)	113 (36.0%)	
PPIs:				<0.001
N	615 (43.6%)	519 (47.4%)	96 (30.6%)	
Y	795 (56.4%)	577 (52.6%)	218 (69.4%)	
Oral antiDM:				0.725
N	783 (55.5%)	605 (55.2%)	178 (56.7%)	
Y	627 (44.5%)	491 (44.8%)	136 (43.3%)	

**Table 2:** The association between the TGI and cardiovascular events.

MACE			
	Yes	No	
Variable	Mean ± STDV	Mean ± STDV	*p*
TGI	9.51 ± 0.66	9.25 ± 0.66	<0.001
Stroke			
TGI	9.42 ± 0.65	9.30 ± 0.67	0.106
CAD			
TGI	9.60 ± 0.67	9.28 ± 0.67	<0.001
CHF			
TGI	9.52 ± 0.71	9.29 ± 0.67	<0.001
MI			
TGI	9.61 ± 0.66	9.27 ± 0.66	<0.001

CAD = coronary artery disease; CHF = congestive heart failure; MACE = major adverse cardiovascular event; MI = myocardial infarction; STDV = standard deviation; TGI = triglyceride–glucose index.

**Table 3:** Regression analysis of the association between the TGI and cardiovascular events.

MACE						
Unadjusted			Partially Adjusted		Fully Adjusted	
Variable	OR (95%CI)	*p*	OR (95%CI)	*p*	OR (95%CI)	*p*
TGI	1.80 (1.48–2.19)	<0.001	1.88 (1.51–2.35)	<0.001	1.80 (1.21–2.69)	0.004
Stroke						
TGI	1.30 (0.95–1.77)	0.105	1.33 (0.95–1.87)	0.097	1.43 (0.78–2.62	0.243
CAD						
TGI	2.01 (1.56–2.60)	<0.001	2.08 (1.56–2.78)	<0.001	2.36 (1.41–3.95)	0.001
CHF						
TGI	1.66 (1.27–2.17)	<0.001	1.67 (1.25–2.23)	<0.001	1.60 (0.95–2.58)	0.076
MI						
TGI	2.09 (1.63–2.69)	<0.001	2.19 (1.65–2.91)	<0.001	2.43 (1.46–4.03)	0.001

Partially adjusted = adjusted for sex, age, body mass index, and smoking status. Fully adjusted = adjusted for partially adjusted + hypertension, creatinine, systolic and diastolic blood pressure, parathyroid hormone, calcium, albumin, HbA1c, low-density lipoprotein, high-density lipoprotein, insulin, aspirin, angiotensin receptor blocker, angiotensin-converting enzyme inhibitor, statin, beta blocker, diuretics, metformin, calcium channel blocker, proton pump inhibitor, and oral anti-diabetic medication. CAD = coronary artery disease; CHF = congestive heart failure; MACE = major adverse cardiovascular event; MI = myocardial infarction; TGI = triglyceride–glucose index.

**Table 4:** Regression analysis for the association between TG-Glu index per quartile and MACE.

MACE						
Unadjusted			Partially Adjusted		Fully Adjusted	
Variable	OR (95%CI)	*p*	OR (95%CI)	*p*	OR (95%CI)	*p*
First quartile	Ref	Ref	Ref	Ref	Ref	Ref
Second quartile	1.44 (0.96–2.18)	0.081	1.49 (0.961–2.29)	0.075	1.88 (0.946–3.73)	0.071
Third quartile	1.93 (1.29–2.87)	0.001	1.95 (1.28–2.97)	0.002	1.88 (1.04–3.73)	0.047
Fourth quartile	2.82 (1.91–4.14)	<0.001	3.16 (2.09–4.78)	<0.001	2.81 (1.34–5.88)	0.006
Stroke						
First quartile	Ref	Ref	Ref	Ref	Ref	Ref
Second quartile	1.39 (0.730–2.63)	0.319	1.43 (0.776–2.63)	0.252	1.54 (0.573–4.12)	0.393
Third quartile	1.39 (0.734–2.65)	0.310	2.00 (1.12–3.57)	0.019	1.19 (0.430–3.27)	0.741
Fourth quartile	1.52 (0.806–2.85)	0.197	3.66 (2.10–6.36)	<0.001	1.68 (0.570–4.95)	0.347
CAD						
First quartile	Ref	Ref	Ref	Ref	Ref	Ref
Second quartile	1.38 (0.760–2.50)	0.291	1.24 (0.681–2.27)	0.480	1.39 (0.506–3.80)	0.525
Third quartile	2.00 (1.14–3.51)	0.015	1.44 (0.800–2.58)	0.225	1.67 (0.646–4.29)	0.291
Fourth quartile	3.17 (1.86–5.40)	<0.001	2.59 (1.49–4.48)	0.001	3.55 (1.32–9.52)	0.012
CHF						
First quartile	Ref	Ref	Ref	Ref	Ref	Ref
Second quartile	1.26 (0.700–2.28)	0.442	1.37 (0.743–2.53)	0.313	1.60 (0.618–4.14)	0.333
Third quartile	1.47 (0.827–2.62)	0.189	1.93 (1.08–3.45)	0.027	1.53 (0.591–3.94)	0.383
Fourth quartile	2.52 (1.48–4.30)	0.001	3.34 (1.91–5.84)	<0.001	2.59 (0.976–6.89)	0.056
MI						
First quartile	Ref	Ref	Ref	Ref	Ref	Ref
Second quartile	1.43 (0.793–2.59)	0.234	1.42 (0.740–2.73)	0.290	1.58 (0.591–4.23)	0.362
Third quartile	2.06 (1.18–3.61)	0.011	1.44 (0.746–2.76)	0.280	1.66 (0.649–4.24)	0.290
Fourth quartile	3.44 (2.02–5.83)	<0.001	1.70 (0.887–3.24)	0.110	3.74 (1.41–9.91)	0.008

Partially adjusted = adjusted for sex, age, body mass index, and smoking status. Fully adjusted = adjusted for partially adjusted + hypertension, creatinine, systolic and diastolic blood pressure, parathyroid hormone, calcium, albumin, HbA1c, low-density lipoprotein, high-density lipoprotein, insulin, aspirin, angiotensin receptor blocker, angiotensin-converting enzyme inhibitor, statin, beta blocker, diuretics, metformin, calcium channel blocker, proton pump inhibitor, and oral anti-diabetic medication. CAD = coronary artery disease; MACE = major adverse cardiovascular event; CHF = congestive heart failure; MI = myocardial infarction.

**Table 5:** ROC analysis results.

MACE				
Variable Cut-Off Point	AUC	*p*	Sensitivity	Specificity
TGI > 9.36	0.61	<0.001	0.57	0.57
Stroke				
TGI > 9.33	0.55	0.141	0.53	0.53
CAD				
TGI > 9.39	0.63	<0.001	0.59	0.58
CHF				
TGI > 9.40	0.6	<0.001	0.57	0.58
MI				
TGI > 9.39	0.64	<0.001	0.59	0.59

CAD = coronary artery disease; CHF = congestive heart failure; MACE = major adverse cardiovascular event; MI = myocardial infarction; ROC = receiver operating characteristics; TGI: triglyceride–glucose index; AUC = area under curve.

**Table 6:** Regression analysis of the association between the TGI and cardiovascular events according to gender and age *.

Sex						
Outcome	Male			Female		
	OR (95%CI)	*p*	FDR-*p*	OR (95%CI)	*p*	FDR-*p*
MACE	2.57 (1.29–5.12)	0.007	0.031	1.98 (1.43–2.53)	0.016	0.044
Stroke	2.47 (1.08–5.65)	0.032	0.063	0.989 (0.01–1.97)	0.982	0.999
CAD	2.12 (0.932–4.82)	0.073	0.362	3.44 (2.62–4.26)	0.003	0.015
CHF	3.06 (1.30–7.18)	0.010	0.046	1.71 (0.906–2.51)	0.187	0.653
MI	1.78 (0.82–3.86)	0.146	0.611	4.21 (3.39–5.03)	0.001	0.006
Age (years)						
Outcome	<60			≥60		
MACE	3.52 (2.58–4.46)	0.036	0.076	1.53 (0.997–2.01)	0.081	0.215
Stroke	3.79 (0.910–6.57)	0.347	0.725	1.68 (0.974–2.39)	0.148	0.620
CAD	5.28 (3.89–6.67)	0.019	0.042	1.91 (1.30–2.52)	0.037	0.061
CHF	4.27 (2.95–5.65)	0.035	0.055	1.36 (0.721–2.00)	0.344	0.888
MI	7.26 (5.83–8.69)	0.007	0.033	1.82 (1.23–2.41)	0.048	0.113

* Data presented from the fully adjusted model = adjusted for sex, age, body mass index, smoking status, hypertension, creatinine, systolic and diastolic blood pressure, parathyroid hormone, calcium, albumin, HbA1c, low-density lipoprotein, high-density lipoprotein, insulin, aspirin, angiotensin receptor blocker, angiotensin-converting enzyme inhibitor, statin, beta blocker, diuretics, metformin, calcium channel blocker, proton pump inhibitor, and oral anti-diabetic medication. CAD = coronary artery disease; CHF = congestive heart failure; MACE = major adverse cardiovascular event; MI = myocardial infarction; TGI: triglyceride–glucose index; FDR = false discovery rate.

## Data Availability

The data presented in this study are available on request from the corresponding author due to privacy and legal reasons.

## Supplementary Materials

The following supporting information can be downloaded at: https://www.mdpi.com/article/10.3390/life15101519/s1, Table S1: Characteristics of the included patients according to their stroke status; Table S2: Characteristics of the included patients according to their CAD status; Table S3: Characteristics of the included patients according to their CHF status; Table S4: Characteristics of the included patients according to their MI status.

## Abbreviations

MACE: Major Adverse Cardiovascular Event, CAD: Coronary Artery Disease, CHF: Congestive Heart Failure, MI: Myocardial Infarction.
