# Supplementary material for: The Association Between the Triglyceride–Glucose Index and the Risk of Cardiovascular Disease in Patients with Type 2 Diabetes Mellitus: A Cross-Sectional Study
Source: Life (Basel). 2025 Sep 26;15(10):1519. doi: 10.3390/life15101519 (PMC12564990; doi:10.3390/life15101519)
Supplement: Supplementary file 1 [file life-15-01519-s001.zip › life-3874251-supplementary.pdf]

Table S1. Characteristics of the included patients according to their stroke status

		Stroke		
		No<br>Count (%)<br>Mean± STDV	Yes<br>Count (%)<br>Mean± STDV	p
Age		59.51±11.19	64.77±9.15	<0.001
Gender	Female	753 (95.68)	34 (4.32)	<0.001
	Male	504 (89.84)	57 (10.16)	
Diabetes Duration		10.35±8	14.27±9.69	<0.001
Smoking status	Non-smoker	895 (94.61)	51 (5.39)	0.002
	Ex-smoker	153 (87.43)	22 (12.57)	
	Smoker	209 (92.07)	18 (7.93)	
Hypertension	No	397 (96.83)	13 (3.17)	0.001
	Yes	860 (91.68)	78 (8.32)	
Body mass index		33.1±6.59	31.37±5.4	0.014
Systolic blood pressure		135.26±21.27	137.79±21.34	0.273
Diastolic blood pressure		77.79±13.5	77.2±12.29	0.683
Creatinine		0.89±0.62	1.15±0.75	<0.001
GFR		99.3±40.27	80.46±37.06	<0.001
CKD score		86.45±26.08	71.84±26.63	<0.001
HbA1c		7.65±1.53	7.74±1.54	0.59
LDL		96.71±35.7	93.43±32.65	0.394
HDL		45.36±21.09	43.17±12.76	0.329
Vitamin d		28.55±17.72	32.94±24.37	0.045
Parathyroid hormone		91.73±90.23	106.15±75.07	0.256
Calcium		9.5±0.52	9.37±0.55	0.023
Phosphorous		3.51±0.77	3.46±0.75	0.573
Albumin		4.29±0.42	4.24±0.41	0.336
Alkaline phosphatase		86.74±57.07	84.75±31.05	0.751
Insulin	No	647 (93.5)	45 (6.5)	0.698
	Yes	608 (92.97)	46 (7.03)	
Aspirin	No	363 (94.78)	20 (5.22)	0.156
	Yes	892 (92.63)	71 (7.37)	
Angiotensin receptor blocker	No	812 (92.69)	64 (7.31)	0.277
	Yes	443 (94.26)	27 (5.74)	
Angiotensin converting enzyme inhibitor	No	1030 (94.41)	61 (5.59)	<0.001
	Yes	225 (88.24)	30 (11.76)	

Statin	No	211 (95.91)	9 (4.09)	0.085
	Yes	1044 (92.72)	82 (7.28)	
Beta Blockers	No	704 (94.24)	43 (5.76)	0.101
	Yes	551 (91.99)	48 (8.01)	
Diuretics	No	804 (94.37)	48 (5.63)	0.031
	Yes	451 (91.3)	43 (8.7)	
Metfomin	No	269 (90.27)	29 (9.73)	0.021
	Yes	986 (94.08)	62 (5.92)	
Calcium channel blockers	No	914 (94.42)	54 (5.58)	0.006
	Yes	341 (90.21)	37 (9.79)	
Proton pump inhibitors	No	546 (94.46)	32 (5.54)	0.121
	Yes	709 (92.32)	59 (7.68)	
Oral anti-diabetic agents	No	681 (92.65)	54 (7.35)	0.348
	Yes	574 (93.94)	37 (6.06)	

CKD=chronic kidney disease; GFR=glomerular filtration rate; HDL=high density lipoprotein; LDL=low density lipoprotein.

Table S2. Characteristics of the included patients according to their CAD status.

		CAD		
		No<br>Count (%)<br>Mean± STDV	Yes<br>Count (%)<br>Mean± STDV	p
Age		59.39±11.21	63.87±9.68	<0.001
Gender	Female	734 (93.27)	53 (6.73)	<0.001
	Male	469 (83.6)	92 (16.4)	
Diabetes Duration		10.13±7.92	14.6±9.2	<0.001
Smoking status	Non-smoker	877 (92.71)	69 (7.29)	<0.001
	Ex-smoker	134 (76.57)	41 (23.43)	
	Smoker	192 (84.58)	35 (15.42)	
Hypertension	No	395 (96.34)	15 (3.66)	<0.001
	Yes	808 (86.14)	130 (13.86)	
Body mass index		32.96±6.58	33.19±6.08	0.678
Systolic blood pressure		135.54±21.45	134.52±19.78	0.592
Diastolic blood pressure		78.04±13.41	75.29±13.26	0.021
Creatinine		0.88±0.62	1.14±0.72	<0.001
GFR		100.3±40.71	79.25±31.29	<0.001
CKD score		86.96±26.08	73.12±25.49	<0.001
HbA1c		7.61±1.53	8.06±1.48	0.001
LDL		97.29±35.65	89.85±33.61	0.017
HDL		46.03±21.39	38.46±10.75	<0.001
Vitamin d		28.9±18.44	28.27±16.49	0.719
Parathyroid hormone		90.42±91.08	109.76±72.77	0.065
Calcium		9.5±0.52	9.42±0.5	0.081
Phosphorous		3.51±0.78	3.44±0.62	0.254
Albumin		4.3±0.4	4.15±0.53	<0.001
Alkaline phosphatase		86.35±57.76	88.69±35.11	0.639
Insulin	No	642 (92.77)	50 (7.23)	<0.001
	Yes	559 (85.47)	95 (14.53)	
Aspirin	No	363 (94.78)	20 (5.22)	<0.001
	Yes	838 (87.02)	125 (12.98)	
Angiotensin receptor blocker	No	790 (90.18)	86 (9.82)	0.123
	Yes	411 (87.45)	59 (12.55)	
	No	984 (90.19)	107 (9.81)	.018*

Angiotensin converting enzyme inhibitor	Yes	217 (85.1)	38 (14.9)	
Statin	No	207 (94.09)	13 (5.91)	.011*
	Yes	994 (88.28)	132 (11.72)	
Beta Blockers	No	721 (96.52)	26 (3.48)	<0.001
	Yes	480 (80.13)	119 (19.87)	
Diuretics	No	791 (92.84)	61 (7.16)	<0.001
	Yes	410 (83)	84 (17)	
Metfomin	No	245 (82.21)	53 (17.79)	<0.001
	Yes	956 (91.22)	92 (8.78)	
Calcium channel blockers	No	869 (89.77)	99 (10.23)	0.302
	Yes	332 (87.83)	46 (12.17)	
Proton pump inhibitors	No	538 (93.08)	40 (6.92)	<0.001
	Yes	663 (86.33)	105 (13.67)	
Oral anti-diabetic agents	No	651 (88.57)	84 (11.43)	0.395
	Yes	550 (90.02)	61 (9.98)	

CKD=chronic kidney disease; GFR=glomerular filtration rate; HDL=high density lipoprotein; CAD=coronary artery disease; LDL=low density lipoprotein.

Table S3. Characteristics of the included patients according to their CHF status.

		CHF		
		No<br>Count (%)<br>Mean± STDV	Yes<br>Count (%)<br>Mean± STDV	p
Age		59.42±11.12	64.26±10.29	<0.001
Gender	Female	724 (92.11)	62 (7.89)	0.013
	Male	494 (88.06)	67 (11.94)	
Diabetes Duration		10.3±7.82	13.6±10.65	<0.001
Smoking status	Non-smoker	864 (91.33)	82 (8.67)	0.008
	Ex-smoker	147 (84)	28 (16)	
	Smoker	207 (91.59)	19 (8.41)	
Hypertension	No	388 (94.87)	21 (5.13)	<0.001
	Yes	830 (88.49)	108 (11.51)	
Body mass index		32.9±6.45	33.72±7.17	0.18
Systolic blood pressure		135.55±20.79	134.51±25.44	0.601
Diastolic blood pressure		77.96±13.26	75.87±14.79	0.096
Creatinine		0.88±0.58	1.22±0.95	<0.001
GFR		100.28±40.18	76.69±35.43	<0.001
CKD score		87.09±25.6	69.97±28.46	<0.001
HbA1c		7.63±1.5	7.93±1.76	0.036
LDL		96.33±35.38	97.99±36.9	0.614
HDL		45.53±21.29	42.23±12.71	0.084
Vitamin d		29.12±18.45	26.2±16.1	0.109
Parathyroid hormone		89.8±89.84	117.18±80.14	0.013
Calcium		9.51±0.52	9.31±0.53	<0.001
Phosphorous		3.51±0.77	3.48±0.71	0.732
Albumin		4.3±0.4	4.13±0.53	<0.001
Alkaline phosphatase		84.47±42.82	106.26±121.26	<0.001
Insulin	No	631 (91.32)	60 (8.68)	0.245
	Yes	585 (89.45)	69 (10.55)	
Aspirin	No	368 (96.34)	14 (3.66)	<0.001
	Yes	848 (88.06)	115 (11.94)	
Angiotensin receptor blocker	No	797 (91.09)	78 (8.91)	0.250
	Yes	419 (89.15)	51 (10.85)	
Angiotensin converting enzyme inhibitor	No	990 (90.83)	100 (9.17)	0.283
	Yes	226 (88.63)	29 (11.37)	

Statin	No	204 (92.73)	16 (7.27)	0.202
	Yes	1012 (89.96)	113 (10.04)	
Beta Blockers	No	715 (95.84)	31 (4.16)	<0.001
	Yes	501 (83.64)	98 (16.36)	
Diuretics	No	805 (94.48)	47 (5.52)	<0.001
	Yes	411 (83.37)	82 (16.63)	
Metfomin	No	259 (86.91)	39 (13.09)	0.020
	Yes	957 (91.4)	90 (8.6)	
Calcium channel blockers	No	886 (91.62)	81 (8.38)	0.016
	Yes	330 (87.3)	48 (12.7)	
Proton pump inhibitors	No	542 (93.93)	35 (6.07)	<0.001
	Yes	674 (87.76)	94 (12.24)	
Oral anti-diabetic agents	No	666 (90.74)	68 (9.26)	0.656
	Yes	550 (90.02)	61 (9.98)	

CKD=chronic kidney disease; GFR=glomerular filtration rate; HDL=high density lipoprotein; CHF=congestive heart failure; LDL=low density lipoprotein.

Table S4. Characteristics of the included patients according to their MI status.

		MI		
		No<br>Count (%)<br>Mean± STDV	Yes<br>Count (%)<br>Mean± STDV	P
Age		59.39±11.24	63.64±9.54	<0.001
Gender	Female	734 (93.27)	53 (6.73)	<0.001
	Male	463 (82.53)	98 (17.47)	
Diabetes Duration		10.1±7.89	14.67±9.25	<0.001
Smoking status	Non-smoker	874 (92.39)	72 (7.61)	<0.001
	Ex-smoker	133 (76)	42 (24)	
	Smoker	190 (83.7)	37 (16.3)	
Hypertension	No	393 (95.85)	17 (4.15)	<0.001
	Yes	804 (85.71)	134 (14.29)	
Body mass index		32.99±6.63	32.94±5.66	0.930
Systolic blood pressure		135.55±21.44	134.52±19.92	0.583
Diastolic blood pressure		78.05±13.43	75.32±13.08	0.020
Creatinine		0.88±0.62	1.12±0.7	<0.001
GFR		100.26±40.81	80.28±31.12	<0.001
CKD score		86.9±26.15	74.08±25.32	<0.001
HbA1c		7.6±1.52	8.08±1.51	<0.001
LDL		97.32±35.67	89.88±33.53	0.015
HDL		46.08±21.42	38.32±10.61	0.001
Vitamin d		28.89±18.47	28.44±16.31	0.001
Parathyroid hormone		90.73±91.26	107.31±72.34	0.799
Calcium		9.5±0.52	9.44±0.5	0.111
Phosphorous		3.52±0.78	3.44±0.62	0.182
Albumin		4.3±0.4	4.16±0.53	0.232
Alkaline phosphatase		86.4±57.88	88.22±34.95	<0.001
Insulin	No	639 (92.34)	53 (7.66)	<0.001
	Yes	556 (85.02)	98 (14.98)	<0.001
Aspirin	No	364 (95.04)	19 (4.96)	<0.001
	Yes	831 (86.29)	132 (13.71)	<0.001
Angiotensin receptor blocker	No	787 (89.84)	89 (10.16)	0.093
	Yes	408 (86.81)	62 (13.19)	
Angiotensin converting	No	978 (89.64)	113 (10.36)	0.038
	Yes	217 (85.1)	38 (14.9)	

enzyme inhibitor				
Statin	No	207 (94.09)	13 (5.91)	0.006
	Yes	988 (87.74)	138 (12.26)	
Beta Blockers	No	719 (96.25)	28 (3.75)	<0.001
	Yes	476 (79.47)	123 (20.53)	
Diuretics	No	786 (92.25)	66 (7.75)	<0.001
	Yes	409 (82.79)	85 (17.21)	
Metfomin	No	244 (81.88)	54 (18.12)	<0.001
	Yes	951 (90.74)	97 (9.26)	
Calcium channel blockers	No	864 (89.26)	104 (10.74)	0.377
	Yes	331 (87.57)	47 (12.43)	
Proton pump inhibitors	No	538 (93.08)	40 (6.92)	<0.001
	Yes	657 (85.55)	111 (14.45)	
Oral anti-diabetic agents	No	648 (88.16)	87 (11.84)	0.430
	Yes	547 (89.53)	64 (10.47)	
CKD=chronic kidney disease; GFR=glomerular filtration rate; HDL=high density lipoprotein; MI=myocardial infarction; LDL=low density lipoprotein.				
